# Novel coronavirus epidemic in the Hungarian population, a cross-sectional nationwide survey to support the exit policy in Hungary

**DOI:** 10.1007/s11357-020-00226-9

**Published:** 2020-07-17

**Authors:** Béla Merkely, Attila J. Szabó, Annamária Kosztin, Ervin Berényi, Andor Sebestyén, Csaba Lengyel, Gergő Merkely, Júlia Karády, István Várkonyi, Csaba Papp, Attila Miseta, József Betlehem, Katalin Burián, Ildikó Csóka, Barna Vásárhelyi, Endre Ludwig, Gyula Prinz, János Sinkó, Balázs Hankó, Péter Varga, Gábor Áron Fülöp, Kornélia Mag, Zoltán Vokó, for the HUNgarian COronaVirus-19 Epidemiological Research (H-UNCOVER) investigators

**Affiliations:** 1 Heart and Vascular Center, Semmelweis University, 68 Városmajor St, Budapest, 1122 Hungary; 2 I. Department of Pediatrics, Semmelweis University, Budapest, Hungary; 3 Clinical Center, University of Debrecen, Debrecen, Hungary; 4 Institute for Health Insurance, Faculty of Health Sciences, Clinical Centre, University of Pécs, 48-as tér 1, Pécs, 7622 Hungary; 5 First Department of Medicine, University of Szeged, Szeged, Hungary; 6 Semmelweis University, Budapest, Hungary; 7 Orthopedic Department, Brigham and Women’s Hospital, Harvard University, Boston, MA USA; 8 Cardiovascular Imaging Research Center, Massachusetts General Hospital, Harvard University, Boston, MA USA; 9 Kenézy Gyula Teaching Hospital, University of Debrecen, Egyetem tér 1, Debrecen, 4032 Hungary; 10 Department of Laboratory Medicine, Clinical Centre, Medical School, University of Pécs, Pécs, Hungary; 11 Institute of Emergency Care and Pedagogy of Health, Faculty of Health Sciences, University of Pécs, Pécs, Hungary; 12 Institute of Clinical Microbiology, Department of Medical Microbiology and Immunobiology, University of Szeged, Szeged, Hungary; 13 Institute of Pharmaceutical Technology and Regulatory Affairs, University of Szeged, Szeged, Hungary; 14 Department of Laboratory Medicine, Semmelweis University, Budapest, Hungary; 15 Department of Infectology, Semmelweis University, Budapest, Hungary; 16 Central Hospital of Southern Pest, National Institute of Hematology and Infectious Diseases, Budapest, Hungary; 17 University Pharmacy Department of Pharmacy Administration, Semmelweis University, Üllői út 26, Budapest, 1085 Hungary; 18 Hungarian Central Statistical Office, Budapest, Hungary; 19 Center for Health Technology Assessment, Semmelweis University, Budapest, Hungary

**Keywords:** SARS-CoV-2, Cross-sectional, COVID-19, Nationwide, Hungary

## Abstract

After months of restrictive containment efforts to fight the severe acute respiratory syndrome coronavirus-2 (SARS-CoV-2) epidemic, European countries are planning to reopen. To support the process, we conducted a cross-sectional survey among the Hungarian population to estimate the prevalence of infectious cases and prior SARS-CoV-2 exposure. A representative sample (*n* = 17,787) for the Hungarian population of 14 years or older living in private households (*n* = 8,283,810) was selected. The study was performed within 16 days after 50 days of restrictions, when the number of confirmed cases was stable low. Naso- and oropharyngeal smears and blood samples were collected for PCR and antibody testing. The testing was accompanied by a questionnaire about symptoms, comorbidities, and contacts. Design-based prevalence estimates were calculated. In total, 10,474 individuals (67.7% taken into account a sample frame error of 2315) of the selected sample participated in the survey. Of the tested individuals, 3 had positive PCR and 69 had positive serological test. Population estimate of the number of SARS-CoV-2 infection and seropositivity were 2421 and 56,439, respectively, thus active infection rate (2.9/10,000) and the prevalence of prior SARS-CoV-2 exposure (68/10,000) was low. Self-reported loss of smell or taste and body aches were significantly more frequent among those with SARS-CoV-2. In this representative, cross-sectional survey of the Hungarian population with a high participation rate, the overall active infection rate was low in sync with the prevalence of prior SARS-CoV-2 exposure. We demonstrated a potential success of containment efforts, supporting an exit strategy. NCT04370067, 30.04.2020.

## Introduction

With the outbreak of the corona virus disease 2019 (COVID-19) pandemic, efforts to estimate the total number infections and to investigate the prior exposure to severe acute respiratory syndrome coronavirus-2 (SARS-CoV-2) are key elements of developing defensive responses. (WHO announces COVID-19 outbreak a pandemic 2020; Guan et al. 2020) International health organizations endorsed general recommendations of strategic preparedness and outlined preventive measures as a part of a response plan in support of all countries. (Coronavirus Disease 2019 (COVID-19) 2020a; Country and Technical Guidance—Coronavirus disease (COVID-19) 2020) Even though these recommendations were incorporated almost universally, each country has experienced a distinct course of the epidemic depending on the timing of safety measure initiation and the degree of compliance with the restrictive measures.

In Europe, the first cluster of cases were confirmed on February 21 in Lombardy, Italy (Onder et al. 2020) (~ 450 miles away from Hungary). As of March 13, Europe was declared to be the active center of the COVID-19 pandemic by the World Health Organization. (Coronavirus disease 2019 (COVID-19) 2020b)

In Hungary, the first two SARS-CoV-2 cases were diagnosed on March 4 (university students who had returned from Asia). By March 11, altogether 16 laboratory-confirmed infections as well as 1 COVID-19–related death were registered. At that time, a state emergency was declared and universities were closed. Subsequently from March 16, further restrictions were introduced, such as prohibition of public gatherings of more than 100 people, closing elementary and high schools, reducing the opening hours of restaurants and cafes, as well as permitting the entry to Hungary of citizens only. Subsequently, on March 28, general lockdown was announced, public events were canceled, and only grocery stores and pharmacies were allowed to remain open; at the time 343 confirmed cases were registered and 11 SARS-CoV-2–related death occurred. Some of the safety measures concentrated only on the most vulnerable segment of the population, the elderly. One example is selective opening hours, providing a period of the day, when the grocery stores, pharmacies, and markets were only open for individuals aged 65 or older.

After around 6 weeks of lockdown, a slow opening of the economy was initiated within countries across Europe. However, in order to safely and effectively execute such a complex process, estimating the total number of infective cases and the prevalence of previous exposure to the pathogen is essential. Investigation of the SARS-CoV-2 virulence and case severity has been extensively studied among patients with severe disease course. (Guan et al. 2020; Onder et al. 2020) However, in order to reveal the full spectrum of disease and adjust public health safety measures accordingly, the rate of mild or asymptomatic infections that do not require medical attention need to be explored. (Lipsitch et al. 2020)

The objective of the present study was to estimate the total number of infectious cases and the prevalence of prior SARS-CoV-2 exposure in the Hungarian population after 50 days of strict containment measures. The study was conducted to support the development of an exit policy from the currently applied safety restrictions.

## Methods

### Study design, patient population

The study was funded by the 2020-2.1.1-ED-2020-00017 grant and was approved by the institutional review board and the local ethics committee (IRB IV/4060-3/2020/EKU).

The target population included individuals aged 14 years or older, living in private households in Hungary. A two-stage stratified probability sample of individuals was selected from the population registry, selecting settlements as primary sampling units (PSU) at the first stage and individuals at the second stage. To obtain equal precision in each region, seven regional samples of equal size were designed. Within each region, the larger settlements as well as settlements with at least five confirmed cases became certainty PSUs. The 181 certainty PSUs cover 55% of the target population and 82% of the overall number of confirmed cases. Within each region, settlements with one to four confirmed cases constituted separate strata and the rest of PSUs were stratified by size, taxable income per capita, and population with tertiary educational level. Overall, 154 strata were defined this way and two PSUs were selected with probability proportional to size within each strata. Altogether, 489 settlements from 3155 were selected. Within the settlements, individuals were selected by systematic random sampling after ordering them by age, as age had been identified as the single most important factor associated with the severity of the infection. A minimum of four individuals were selected from each selected settlement. The total number of the sample size was determined by assuming 10% sampling frame error and 70% participation rate. Thus, 17,787 individuals were selected to ensure a planned effective sample size of 11,206. To ensure the temporally cross-sectional nature of the study, the data collection period was restricted to 16 days that fell under the same restriction regulations starting on May 1.

**Fig. 1:**
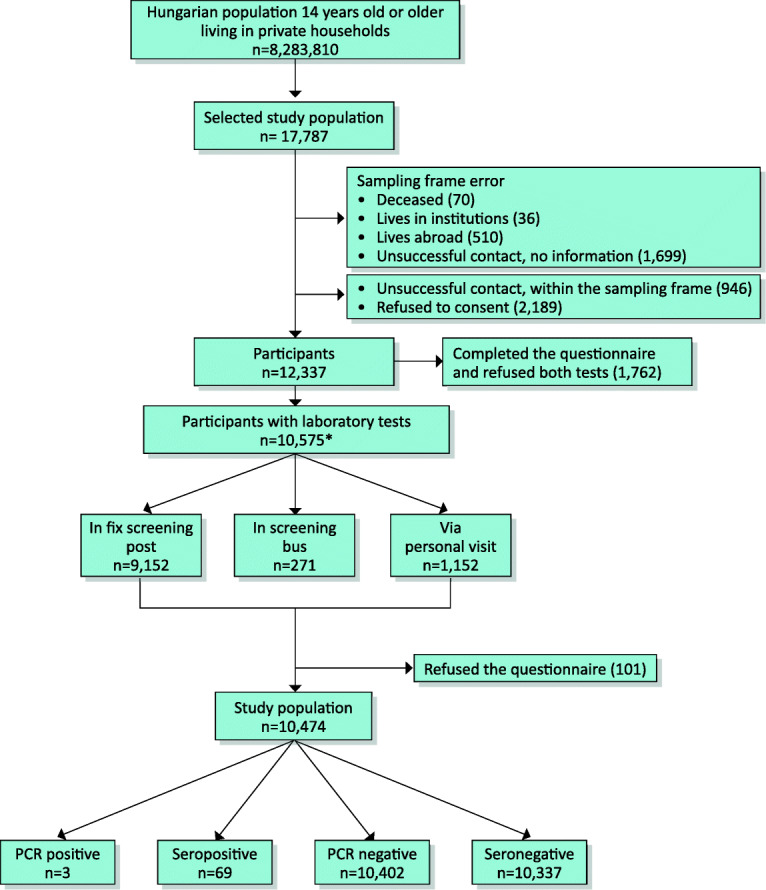
Formulation of the study population. *The sum of the number of positive and negative tests do not add up to the number of the study population (10,474) neither for PCR nor for serology as some persons consented to provide only one type of samples

Those participants were included in the final study population who had either PCR test or serological testing with a completed questionnaire (Fig. 1).

### Contacting selected individuals

We informed selected individuals with an official invitation letter via mail. Those who had previously authorized to be contacted officially by e-mail received an additional invitation electronically. In addition, 14,250 participants with a telephone number registered in the administrative databases were contacted by phone by the Central Statistical Office and the universities. Almost 3000 general practitioners and the local municipalities provided help to contact and motivate the selected participants. Finally, those who we failed to be contacted were visited on their registered address.

### Screening process and sample collection

Screening was carried out as a collaborative effort of four medical universities across Hungary (Semmelweis University, University of Debrecen, University of Szeged, University of Pécs) and the Hungarian Central Statistical Office. Individuals selected for screening were required to register online or over the phone through a dedicated line. The samples were collected by 187 screening teams in 348 fixed screening posts, in five screening buses, and by mobile testing units through a personal visit to those who could not be mobilized. These efforts were supported by the ambulance service, municipalities, and local governmental offices.

### Laboratory measurements

#### PCR test

To detect or exclude the presence of SARS-CoV-2 virus, nasopharyngeal and oropharyngeal samples were collected in viral transport medium tubes and were transported to the laboratory at 2–8 °C. After nucleic acid extraction, real-time PCR was performed (HBRT-COVID-19; Chaozhou Hybribio Biochemistry Ltd., Chaozhou, Guangdong, China) (Organization WH 2020). PCR was performed within 24 h after sample collection. The test used detects the presence of two SARS-CoV-2 viral genes and also applies a human gene sequence as an internal quality control for sampling. The absence of amplified human gene in PCR product indicates inappropriate sampling. Patients with inappropriate samples were re-sampled the day after and provided samples that were appropriate for testing.

Those who tested positive were informed within 2 days and were required to self-isolate for 2 weeks. Positive test results were also reported toward the Hungarian Health Authorities. Participants had access to their own results via the National eHealth Infrastructure.

#### Blood sample analysis

For serological testing, blood samples were obtained from all participants at the age of 18 or older; under 18 years of age, blood testing was optional. Serological testing for SARS-CoV-2–specific IgG was analyzed with commercially available Food and Drug Administration–approved immunoassay (SARS-CoV-2 IgG Reagent Kit Cat. No. 6R86-32 on Architect i2000SR instruments; Abbott Laboratories, Irving, TX, USA), (Abbott 2020) and the remnant samples were stored at − 80 °C.

#### Questionnaire

All participants were invited to fill out a questionnaire during the registration process online, over the phone, or during the screening in person. The questionnaire contained questions regarding socio-economic status, risk factors (smoking and body mass index—BMI), comorbidities, adherence to safety measures, recent travel history, history of symptoms suggestive for COVID-19, and history of a known contact with a confirmed SARS-CoV-2–infected individual or with a person in quarantine.

#### Definition of confirmed cases

To provide a comprehensive background of the COVID-19 epidemic in Hungary, beyond the results of this current survey, we report the age, sex, and regional distribution of all reported confirmed cases and COVID-19–related mortality registered in Hungary until May 16 according to the National Public Health Center. We provide this data for individuals at the age of 14 years or older, living in private households and separately for those living in institutions (homeless shelter, long-term care facility or nursery home).

#### Statistical analysis

We estimated the population prevalence of acute infection and seropositivity by age, sex, and region, categories of labor activity, contact with a person infected with SARS-CoV-2 or being in quarantine, and by visiting a foreign country since March 1, 2020.

To reduce bias in weighting, we could use several area-, dwelling unit-, and individual-level auxiliary information from sampling frame and other administrative data sources, each related to both non-response and the objective variable. After adjusting design weights, the response sample was calibrated to known population counts by region, sex, and age categories.

Variance estimation method took calibration effect as well as stratification and clustering into account. For the residuals by calibration variables, we used Taylor-linearized variance estimation. (Wolter 2007) In case of zero observation in a subgroup, we used the rule-of-three method to estimate confidence intervals (i.e., 95% confidence interval of the prevalence was estimated as 0–3/*n*). (Eypasch et al. 1995) The calculations were performed using SAS software version 9.4.

## Results

**Fig. 2:**
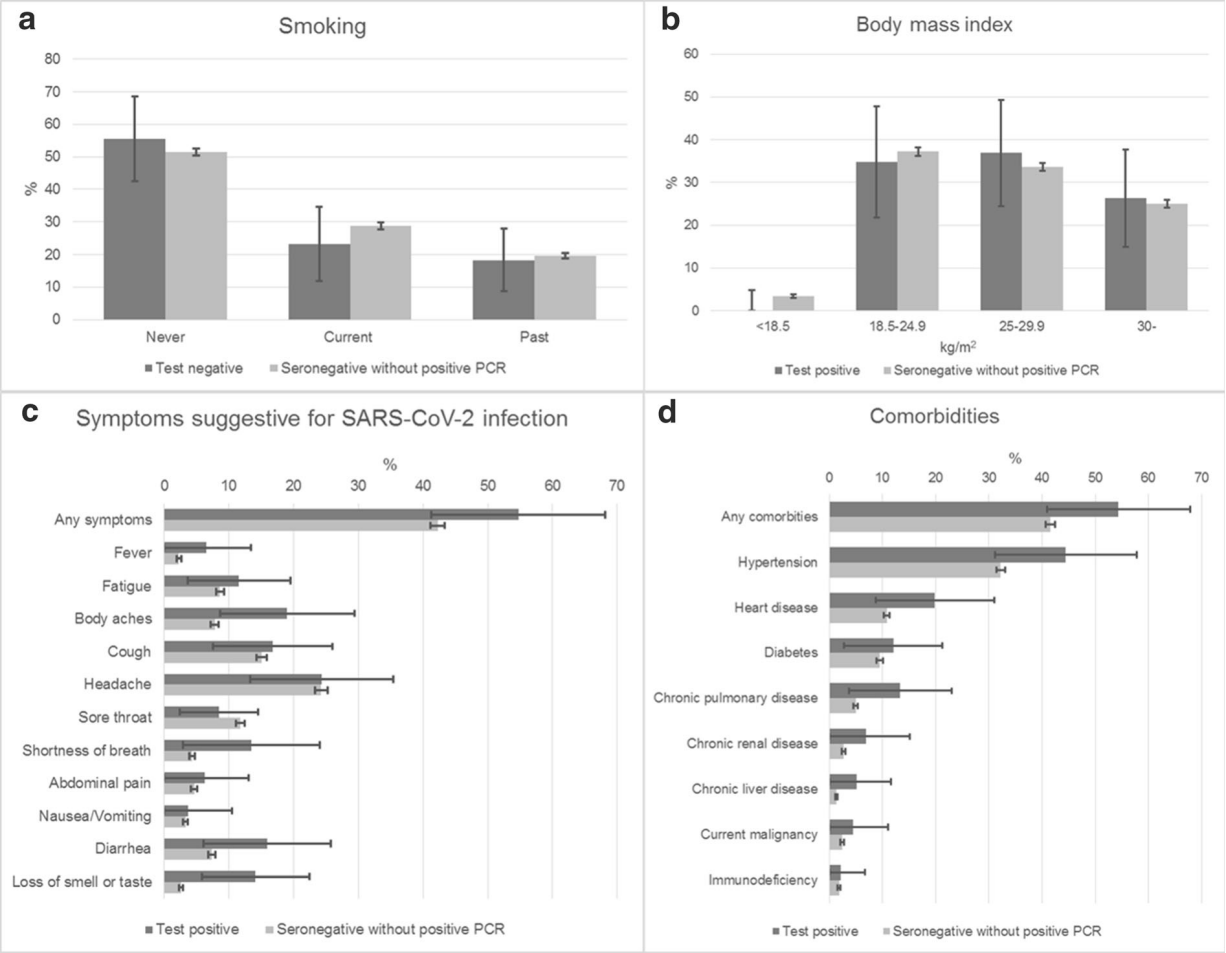
Population estimates of the distribution of smoking (**a**), body mass index (**b**), symptoms (**c**), and comorbidities (**d**) by infection

Of the planned calculated sample of 17,787 individuals, 10,505 underwent PCR testing and 10,504 had a serological test, while 10,434 had both. In total, 12,236 individuals completed the questionnaire. Altogether, 10,474 people, 67.7% of the 15,472 individuals belonging to the sampling frame, who had either a PCR or a serological test with a completed questionnaire, were included into our final study population (Fig. 1). The mean age was 48.7 years and the 46.4% were male (Table 1). The population estimate of the proportion of individuals who experienced any symptoms suggestive for SARS-CoV-2 infection was larger among those with a positive PCR or immunological test compared with seronegative persons: 54.7% vs. 42.2% (Fig. 2c). Body aches and loss of smell or taste occurred significantly more frequently among people with a positive test (19.0%, 95% CI 8.6–29.3% vs. 7.8%, 95% CI 7.2–8.4%, and 14.1%, 95% CI 5.8–22.4 vs. 2.6%, 95% CI 2.3–2.9%, respectively). Shortness of breath and diarrhea was also a common symptom in the seropositive group, although compared with the seronegatives, the difference was not statistically significant. The estimated proportion in the population of people who had any comorbidities was higher among persons with a positive test: 54.4% vs. 41.5% (Fig. 2d).

**Table 1:** Characteristics of the study population

	Total study population (*n* = 10,474)	Test positive* (*n* = 70)	Seronegative without positive PCR (*n* = 10,336)	Negative PCR test without serology (*n* = 68)
Men (%)	4864 (46.4)	35 (50.0)	4798 (46.4)	31 (45.6)
Age (years)				
Mean (SD)	48.7 (18.0)	52.2 (18.2)	48.7 (18.0)	45.1 (21.3)
14–39 (%)	3353 (32.0)	18 (25.7)	3309 (32.0)	26 (38.2)
40–64 (%)	4735 (45.2)	33 (47.1)	4676 (45.2)	26 (38.2)
65– (%)	2386 (22.8)	19 (27.1)	2351 (22.7)	16 (23.5)
BMI (kg/m^2^)†				
<18.5	322	0	315	7
18.5–24.9	3709	21	3662	26
25–29.9	3589	27	3546	16
30–	2774	20	2736	18
Smoking†				
Never	5381	38	5310	33
Current	2933	16	2904	13
Past	2143	15	2107	21
Any symptoms after 03.01.2020‡	4470	37	4406	27
Fever	239	3	233	3
Fatigue	883	9	868	6
Body aches	832	12	817	3
Cough	1630	13	1609	8
Headache	2548	19	2513	16
Sore throat	1290	8	1276	6
Shortness of breath	432	6	425	1
Abdominal pain	471	4	467	0
Nausea/vomiting	333	1	332	0
Diarrhea	773	10	758	5
Loss of smell or taste	277	12	261	4
Reported any comorbidities‡	4533	38	4461	34
Hypertension	3549	32	3489	28
Heart disease	1135	11	1115	9
Diabetes mellitus	1059	7	1045	7
Chronic pulmonary disease	522	8	505	9
Chronic renal disease	262	3	256	3
Chronic liver disease	141	3	137	1
Current malignancy	262	2	256	4
Immunodeficiency	201	1	200	0

The relative frequencies of men and age categories in the population are the same as in the sample, as the weights were calibrated to these characteristics. The population estimates of the other relative frequencies are presented in Fig. 2

*Either with positive PCR or positive serological test

†As some of the participants did not answer the question, the percentages do not add up to 100%

‡More than one category could be indicated

### Test results in the studied population

Three participants had a positive PCR test; two of them were hospitalized due to confirmed COVID-19 infection. Altogether, 70 individuals had a positive serological test result, 69 with a completed questionnaire. Of the seropositive individuals, two had a simultaneous positive PCR test.

### Estimated population infection rate

Based on the results of the survey, the estimated number of PCR-positive and seropositive individuals in the entire Hungarian population 14 years or older who live in independent households (*n* = 8,283,810) were 2421 and 56,439, respectively.

The estimates in Table 2 suggest that seropositivity tends to increase by age (between 14 and 39 years—56/10,000 vs. 65 years or older—83/10,000) and is higher among those who commuted regularly to their workplace (commutes several times—59/10,000 vs. worked from home—42/10,000), had a contact with confirmed SARS-CoV-2–infected individual or someone in quarantine (individuals with a history of contact—114/10,000 vs. without a contact—66/10,000), and traveled abroad after March 12, 2020 (traveled abroad—106/10,000 vs. no travel—67/10,000). However, none of these differences were statistically significant.

**Table 2:** Population estimates of PCR-positive and seropositive cases

	PCR positive		Seropositive	
	Estimated total number	Prevalence per 10,000 (95% CI)	Estimated total number	Prevalence per 10,000 (95% CI)
Total	2421	2.9 (0–6.7)	56,439	68 (50–86)
Men	713	1.8 (0–5.8)	27,323	70 (44–95)
Women	1708	3.9 (0–10)	29,115	67 (42–92)
Age (years)				
14–39	0	0 (0–8.9)	16,637	56 (27–86)
40–64	1269	3.7 (0–11)	24,127	70 (44–96)
65–	1152	6.1 (0–16)	15,674	83 (39–126)
Labor activity				
Active worker	1269	3.0 (0–9.0)	22,406	53 (33–74)
Pensioner	1152	5.4 (0–14)	23,412	109 (66–152)
Student, not working	0	0 (0–38)	4932	69 (0–149)
Housewife	0	0 (0–142)	1239	73 (0–174)
Other non-worker	0	0 (0–27)	4450	43 (1.6–84)
Place of work during the epidemic*				
Commutes several times a week	1269	4.3 (0–13)	17,329	59 (33–84)
Commutes once a week	0	0 (0–93)	948	40 (0–130)
Home based	0	0 (0–25)	4128	42 (7.7–76)
Known contact with a confirmed SARS-CoV-2–infected person or a person being in quarantine				
Yes	0	0 (0–73)	3386	114 (24–204)
No	2421	3.1 (0–7.0)	52,044	66 (48–84)
Refused to answer	0	0 (0–252)	1009	106 (0–286)
International travel after 03.01.2020				
Yes	0	0 (0–79)	3460	106 (12–200)
No	2421	3.1 (0–7.0)	52,979	67 (49–86)

*Only among active workers

**Fig. 3:**
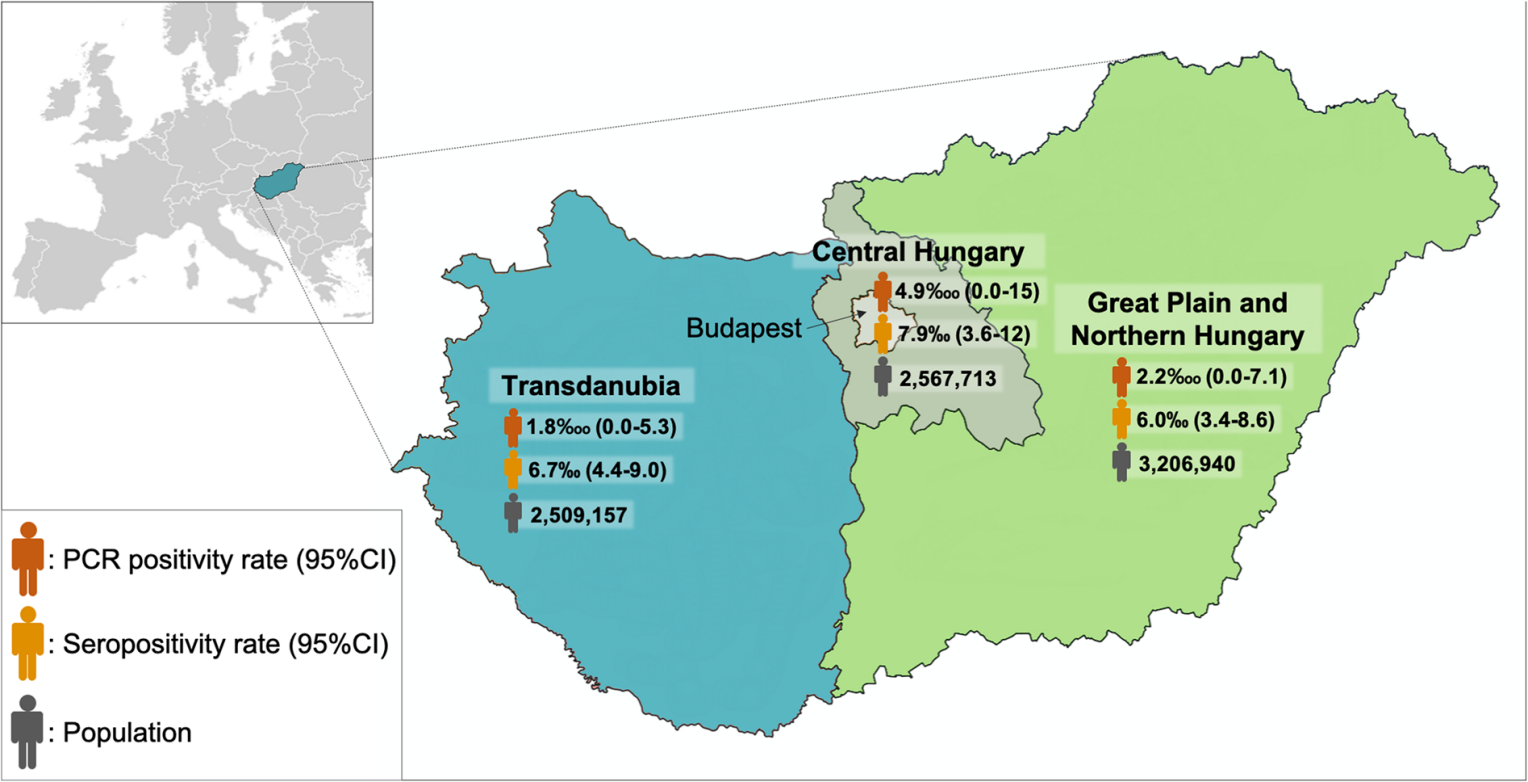
Estimated number and PCR positivity and seropositivity by statistical region

By large statistical region, the highest prevalence of seropositivity was found in the central region including the capital, Budapest (Fig. 3). By subregion, the difference was larger between Budapest (90/10,000) and the two least developed regions: Southern Transdanubia (46/10,000) and Northern Hungary (45/10,000) (Table 3).

**Table 3:** Regional distribution of the target and the study population, and estimated PCR-positive and seropositive cases by region

			PCR positive		Seropositive	
Region	Total population	Sample size	Estimated total number	Prevalence per 10,000 (95% CI)	Estimated total number	Prevalence per 10,000 (95% CI)
Central						
Budapest	1,480,190	987	1269	8.6 (0–26)	13,393	90 (29–152)
Pest county	1,087,523	768	0	0 (0–39)	6920	64 (9.4–118)
Transdanubia						
Central Transdanubia	907,796	1606	439	4.8 (0–15)	6147	68 (29–107)
Western Transdanubia	853,686	1480	0	0 (0–20)	7215	85 (40–129)
Southern Transdanubia	747,675	1335	0	0 (0–22)	3465	46 (14–79)
Great Plain and Northern Hungary						
Northern Hungary	942,357	1458	713	7.6 (0–24)	4236	45 (4.6–85)
Northern Great Plain	1,212,205	1406	0	0 (0–21)	8489	70 (22–118)
Southern Great Plain	1,052,378	1434	0	0 (0–21)	6574	62 (22–103)

The total number of the reported confirmed cases by the National Public Health Center in Hungary until the end of the study was 3464, of whom 2580 lived in private households and were 14 years or older. In the latter group, the number of confirmed new cases during the study period of May 1–16 ranged between 15 and 56, an average 27 per day. The number of registered deaths was 350 and 101 among persons living in private households and in institutions, respectively. The age distribution of the confirmed cases reported until May 16 living in private households was similar to the age distribution of the test-positive subjects identified in the survey (Table 4). The regional variation was larger in the confirmed cases than in seropositive ones: among those who live in private households, 61% of the confirmed cases occurred in the Central region, whereas seropositive individuals were distributed evenly by large statistical region (Tables 3 and 4). The sex and regional distributions of the confirmed cases were identical among those who lived in private households and in institutions. On the other hand, the proportion of elderly people was twice as large in the latter group than in the former.

**Table 4:** Characteristics of the confirmed 14 years old and older COVID-19 cases reported until May 16, 2020 in Hungary

	Living in private households	Living in institutions
Men	1080 (41.9)	360 (40.7)
Women	1500 (58.1)	524 (59.3)
Region		
Central	1583 (61.4)	547 (61.9)
Transdanubia	742 (28.8)	254 (28.7)
Great Plain and Northern Hungary	255 (9.9)	83 (9.4)
Age (years)		
14–39	525 (20.4)	50 (5.7)
40–64	1090 (42.3)	178 (20.1)
65–	965 (37.4)	656 (74.2)

The numbers in brackets are column percentages

## Discussion

In the H-UNCOVER representative, cross-sectional population survey of Hungarian individuals, we investigated the total number of active infections and prevalence of virus exposure after 50 days of the initiation of containment regulations. We provide the first comprehensive analysis utilizing PCR and serological testing simultaneously. Of the selected representative population sample, 67.7% was tested and completed the questionnaire resulting in a considerably high participation rate. Despite the close proximity of major infectious clusters, due to the early introduction of containment efforts, the overall active infection rate remained low (2.9/10,000), in sync with the prevalence of SARS-CoV-2 exposure (68/10,000).

The relatively higher rate of participation in comparison with similar studies conducted to describe the epidemic of SARS-CoV-2 (Gudbjartsson et al. 2020) could be attributed to multiple factors. First, in order to mobilize as many participants as possible, we contacted individuals on three parallel levels: each person was notified via mail and over the phone; as well as general practitioners from around the country contacted selected individuals from their practice. Second, investigators of our study were supported by local governors and the media to spread information to reach out to as many individuals as possible. Third, to enhance the accessibility of testing units, we established even distribution of designated testing facilities and mobile testing units across the country. In addition, we provided in-person visits for two reasons: to further improve the study participation and to test individuals who were otherwise not mobilizable. Lastly, the availability of participants in their homes was considerably higher due to the quarantine and travel restrictions.

In this survey, a relatively low active SARS-CoV-2 infection rate (0.029%) and in sync low overall seropositive rate was identified (0.68%). In Hungary, 12 days after the first SARS-CoV-2–infected cases were confirmed, restrictive measures were implemented. The authors believe that the early initiation of strict containment efforts may explain the control of the spread of SARS-CoV-2 infection. It is important to underline that there were also specific safety measures in Hungary solely applied to elderly people, such as specific opening hours when only the elderly population could visit markets, grocery stores, or pharmacies. In addition, the adherence of the Hungarian population to these regulations also has to be emphasized. Data from different sources including online surveys, depersonalized aggregated mobile cell and traffic data showed a drastic, 60–90% reduction in the number of contacts and extreme reduction in mobility. (Röst et al. 2020) It has been shown with the use of mobility and COVID-19 epidemiology data from the European countries that successful restrictive policy can significantly contribute to the suppression of the SARS-CoV-2 pandemic. (Vokó and Pitter 2020)

Furthermore, consistently with the low level of seropositivity reported in our study, the rate of incident cases registered by the National Public Health Center in Hungary throughout the timeframe of the testing was low as well. However, besides the early containment efforts, there have been a few additional potential protective factors suggested against COVID-19, such as BCG vaccination or blood type 0 which is representative in the Hungarian population. (Curtis et al. 2020; Zhao et al. 2020) In Hungary, BCG vaccination is mandatory, and type 0 is relatively frequent, thus the population may have lower susceptibility to SARS-CoV-2; however, more robust evidence needs to be provided to prove the protective effect of these factors.

The proportion of reported confirmed cases was substantially higher in the Central region of Hungary than the proportion of seropositivity as identified via the cross-sectional survey, which can be explained by the higher rate of testing performed in this area: among those with a PCR test performed until May 16, 43% was obtained in the Central region, which represents 30% of the total population.

Our results suggest a higher SARS-CoV-2 infection rate in older individuals and with persons with chronic diseases, which have been shown to be a risk factor for more severe disease course. (Grasselli et al. 2020) We must also note that a large proportion of reported cases occurred in institutions (Table 4), mainly in nursing homes. Among them 74% were 65 years or older, and mortality was much higher among elderly people. (Kemenesi et al. 2020) The reason for this might be that in older individuals, the immune response is less effective. Moreover, there is evidence that in older individuals suffering from SARS-CoV-1 infection, the switch from innate to adaptive immunity is impaired resulting in an insufficient antibody production, which seems to be the case in SARS-CoV-2 infection as well. (Nikolich-Zugich et al. 2020) This highlights the need for specific interventions and safety measures in elderly care facilities as part of the exit strategy, such as prolonged restrictive measures including strict visitor policy, rigorous cleaning and disinfection protocols, mandatory use of personal protective equipment by the staff, appropriate training of the personnel, viral testing of the new residents, frequent surveillance of symptoms suggestive for SARS-CoV-2, and adequate policies and organization to isolate individuals with suspected infection.

In line with prior evidence, our data also showed that loss of smell or taste might be associated with SARS-CoV-2 infection. (Spinato et al. 2020) We also found that diarrhea was more common among seropositive people, which is in accordance with the previously reported Italian and Chinese data. (Lin et al. 2020; Buscarini et al. 2020)

Based on the results reported in the present study exploring the epidemiology of SARS-CoV-2 exposure, the development of an exit strategy from the currently applied containment regulations is feasible. General regulations considered to be essential to control the spread of SARS-CoV-2, which were introduced all over the world, were adapted in Hungary in a relatively early phase. Due to differences in the implementation of safety recommendations, however, each country may have its own characteristic course of the epidemic. Therefore, we believe that each country needs to survey their population in order to learn their unique characteristic of COVID-19 epidemic and to develop their individual strategic plan to establish a transition back to normal everyday living and to resuscitate the economy. We acknowledge that a sensitive balance between people’s health and economy exists; however, an early opening of the economy might undermine positive effects of restrictive efforts. Our study suggests that early initiation of safety measures and adherence to regulations can decrease the spread of SARS-CoV-2 and result in low COVID-19–related morbidity and mortality, especially protecting the elderly (aged 65 and older) who are the most vulnerable against the SARS-CoV-2 infection.

Results of the present study mirror the situation after a time period of 50 days of safety restrictions. In order to track the effect of economic reopening and loosening of restrictive safety measures, repeating this representative population-based cross-sectional survey with a nested longitudinal follow-up of a subgroup is planned. Moreover, as future waves of COVID-19 are predicted, until herd immunity is not obtained or until large-scale vaccination cannot be maintained, the need for such cross-sectional surveys are even more pronounced.

Our survey had certain limitations. First, selective non-response could be considered as a potential limitation; however, it is very unlikely that the participation in the study was related to a previously undiagnosed SARS-CoV-2 infection. Second, while PCR testing is regarded as the current standard diagnostic method for SARS-CoV-2 infection, the risk of false negativity can be significant and could be decreased significantly with repeated sampling which was not feasible in such a study.

In conclusion, our study suggests that early initiation of containment efforts and adherence to regulations may decrease the spread of SARS-CoV-2 and could result in low COVID-19–related morbidity and mortality. Consequently, an exit strategy from the currently applied containment regulations is feasible.
